# Quality of life and survival in patients with uterine carcinosarcoma: A tertiary center observational study^☆^

**DOI:** 10.1016/j.gore.2025.101679

**Published:** 2025-01-17

**Authors:** Eveline N.B. Pham, Caroline B. van den Berg, Ingrid Boere, Vera de Geus, Juanita A. Haagsma, Marianne Maliepaard, Jan Willem M. Mens, Floris H. Groenendijk, Heleen J. van Beekhuizen

**Affiliations:** a Department of Gynecologic Oncology, Erasmus MC Cancer Institute, University Medical Center Rotterdam, Molewaterplein 40, 3015 GD Rotterdam, the Netherlands; b Department of Medical Oncology, Erasmus MC Cancer Center, Molewaterplein 40, 3015 GD Rotterdam, the Netherlands; c Department of Public Health, Erasmus MC University Medical Center, Molewaterplein 40, 3015 GD Rotterdam, the Netherlands; d Department of Radiotherapy, Erasmus MC Cancer Institute, Molewaterplein 40, 3015 GD Rotterdam, the Netherlands; e Department of Pathology, Erasmus MC Cancer Institute, Molewaterplein 40, 3015 GD Rotterdam, the Netherlands

**Keywords:** Uterine carcinosarcoma, Endometrial cancer, Quality of life, Survival

## Abstract

**Objective:**

This study aimed to assess the QoL in patients with uterine carcinosarcoma (UCS) and identify factors influencing survival.

**Methods:**

A prospective cohort study was conducted of patients with UCS who were treated between 2016 and 2022 in a Dutch academic hospital. QoL was evaluated using the EORTC QLQ-C30 and QLQ-EN24 questionnaires at baseline, end of treatment, and at one, two, and five years after treatment. Outcomes were described as mean scores in functioning and symptom domains. The mean scores of the QLQ-C30 were compared to those of a matched group from the general Dutch population. Clinical data were collected. Factors influencing survival were assessed via Cox proportional hazards models.

**Results:**

The study included 64 patients, 56 of them participated in the QoL questionnaires. The median progression-free survival was 13 months, and overall survival was 20 months. QoL was significantly lower in all functional domains at one year after treatment compared to members of the general population. The most affected functioning domains were physical, role, cognitive, and social functioning and symptoms like fatigue, pain, and lymphedema. Long-term QoL remained compromised, with global health and insomnia notably impaired even five years after treatment. Significant prognostic factors for better overall survival included lower FIGO stage, surgery, and chemotherapy.

**Conclusions:**

Patients with UCS experience significant deterioration in QoL. Despite extensive treatment, QoL remains poor, emphasizing the need for integrating QoL considerations into treatment decisions to balance the benefits of treatment with potential QoL impairments.

## Highlights

• Uterine carcinosarcoma has a poor survival with 5-year survival of 30–45% for early stage and 0–10% for advanced stage.
• Quality of life of patients with uterine carcinosarcoma is significantly reduced by disease and treatment factors.
• This underscores the need to consider quality of life into treatment decisions to balance benefits with harms.

## 1 Introduction

Uterine carcinosarcoma (UCS) is a high-grade endometrial cancer, which accounts for approximately 5 % of all endometrial cancers (Nama et al., 2020, Bogani et al., 2023). With a 5-year overall survival of 30–45 % for early stage and 0–10 % for advanced stage disease, it has a lower survival compared to other high-grade endometrial cancers, such as serous and clear cell carcinomas (Nama et al., 2020, Concin et al., 2021, Raffone et al., 2022, de Jong et al., 2011, Zhang et al., 2015). Surgical treatment is similar as for other high-grade endometrial cancer, including staging hysterectomy, bilateral salpingo-oophorectomy and pelvic and *para*-aortic lymphadenectomy for early-stage disease and cytoreductive surgery for advanced stages (Bogani et al., 2023, Concin et al., 2021, Abu-Rustum et al., 2024, PDQ, 2024). Adjuvant chemo- and radiotherapy are recommended based on the surgical stage and associated risk factors. The prognosis remains poor despite extensive treatment (Nama et al., 2020, Bogani et al., 2023, Concin et al., 2021), while treatment is not harmless. This is not only due to the risk of adverse events, but also because treatment is likely to impact the quality of life (QoL) of patients, which may already be diminished by the disease itself (Shisler et al., 2018, Gil-Ibanez et al., 2023). In other types of high-grade endometrial cancer QoL often returns to baseline within four to six months after treatment, (Shisler et al., 2018, Gil-Ibanez et al., 2023). However, QoL in specifically UCS has not been studied yet, while prioritizing QoL for patients with UCS is especially important given the limited life expectancy. Previous studies on various types of advanced-stage cancers have shown that over half of patients prefer better QoL over extended survival when considering treatments (Kypriotakis et al., 2016, Brundage et al., 2023, Fleischer et al., 2023, Yong et al., 2023).

Hence, shared decision making in treatment of UCS is important, taking not only the benefit of treatment into consideration, but also the QoL. Whereas clinical outcomes of patients with UCS have been described before in observational studies (Can et al., 2023, Aroche Gutierrez et al., 2024, Cantrell et al., 2012, Matsuo et al., 2018, Matsuo et al., 2016, Rosati et al., 2023), impact of treatment on QoL of patients with UCS has not been studied yet. Therefore, we assessed the QoL in patients with UCS up to five years after treatment and disclosed prognostic factors of survival.

## 2 Material & Methods

### 2.1 Study design and patient population

This is a prospective cohort study in patients with UCS treated between January 2016 and December 2022 at Erasmus Medical Center in Rotterdam, The Netherlands, with approval from the Medical Research Ethics Committee (MEC-2015–417). Patients with histologically confirmed UCS were eligible. For the QoL assessment, patients needed to comprehend the questionnaires in Dutch or English. Only clinical data were used of patients who consented to use their data but did not want to participate in QoL questionnaires. Written consent was obtained prior to study participation.

### 2.2 Clinical data collection

Demographic and clinical data were collected from the electronic medical record using a standardized case report form. Patients were staged according to the International Federation of Gynecology and Obstetrics (FIGO) staging system 2009 for endometrial cancer (Creasman, 2009) Staging surgery included hysterectomy, bilateral salpingo-oophorectomy, omentectomy, peritoneal biopsies and pelvic and *para*-aortal lymphadenectomy following international guidelines (Bogani et al., 2023, Concin et al., 2021, Abu-Rustum et al., 2024, PDQ, 2024);supplementary table 1). Side-effects and complications during and after treatment were also recorded. Survival was determined as progression-free survival and overall survival. Progression-free survival was defined as the interval from the date of the first biopsy showing UCS to date of disease progression based on radiological and/or histological findings, or date of death unrelated to UCS. Overall survival was defined as the interval as the interval from the date of the first biopsy showing UCS to date to death (any reason) or 30th April 2024 if the patient was still alive at that time. Median follow-up time of censored patients were calculated from the as the interval from the date of the first biopsy showing UCS until last visit or death unrelated to the UCS.

### 2.3 QoL data collection

QoL was assessed using the EORTC QLQ-C30 and EORTC QLQ-EN24, designed to respectively evaluate QoL in cancer patients in general and in endometrial cancer patients specifically. Questionnaires were obtained at baseline (moment of diagnosis), end of treatment, and one, two, and five years after treatment. Non-responders for a specific time point continued to receive questionnaires for future assessments. Reasons for non-responders were unknown.

Following the EORTC scoring manual, scores for functioning domains (higher scores = better QoL) and symptom domains (higher scores = worse symptoms) were calculated on a 0–100 scale. QLQ-C30 assessed five functioning (Global health status, Physical, Role, Emotional, Cognitive) and nine symptom domains (Fatigue, Nausea/vomiting, Pain, Dyspnea, Insomnia, Appetite loss, Constipation, Diarrhea, Financial problems). The QLQ-EN24 covered three symptom (Sexual interest, Sexual activity, and Sexual enjoyment) and eleven functioning domains (Lymphedema, Urological, Gastrointestinal, Body image, Sexual/vaginal, Back/pelvic pain, Tingling/numbness, Muscular pain, Hair loss, Taste changes, and Hormonal). The QLQ-C30 scores were compared with normative data from Dutch general population (de Ligt et al., 2023), matched by age category and gender for each participant in the QoL questionnaires.

### 2.4 Statistical analysis

SPSS Statistics Version 28.0.1.0 was used for all analyses. Descriptive statistics were used for clinical characteristics. Means and standard deviations (SD) were calculated for the QoL-summary score. T-test was used to compare the outcomes of the questionnaires by our cohort with the normative data, using the reported mean and standard deviation (SD) by De Ligt. (de Ligt et al., 2023) No adjustments were made for patients who had died in the follow-up regarding the QoL-measurements. Univariate and multivariate Cox proportional regression analyses were performed to evaluate independent predictors (age, WHO performance status, moment of diagnosis, FIGO stage, multiple histological characteristics, and treatment) of overall survival and to estimate hazard ratios (HRs). Factors with a p-value ≤ 0.1 on univariate analysis were included in multivariate analyses. In accordance with the journal’s guidelines, we will provide our data for independent analysis by a selected team by the Editorial Team for the purposes of additional data analysis or for the reproducibility of this study in other centers if such is requested.

## 3 Results

In total 64 patients were included in this study, 56 of them participated with the QoL questionnaires. Median follow up time of the censored patients was 68 months (range 18–99 months). Table 1 summarizes baseline characteristics. The mean age at diagnosis was 69, 38 % had a prior cancer history, predominantly breast cancer (63 %), with seven using tamoxifen. Most cases were at clinical stage I (65.6 %), with one case stages II (1.6 %), seven stage III (10.9 %), and 14 stage IV (21.9 %).

**Table 1:** Baseline Characteristics.

**Characteristic**	**N = 64**	**%**
Mean age in years (range)	69 (50–86)	

WHO performance status		
0	43	67.2
1	13	20.3
2	4	6.3
3	3	4.7
Unknown	1	1.6

Race		
Whites	52	81.3
Blacks	9	14.1
Asians	3	4.7
Mean BMI in kg/m^2^ (range)	29 (16–42)	
Underweight (<18.5)	3	4.7
Normal BMI (>18.5 and < 25)	14	21.9
Overweight (>25 and < 30)	25	39.1
Obese (>30)	21	32.8
Unknown	1	1.6

Parity		
0	9	14.1
1	8	12.5
2-3	41	64.1
4+	6	9.4
Malignancy in medical history	24	37.5
Breast cancer	15*	23.4
Urologic cancer	1*	1.6
Colorectal cancer	1*	1.6
Other†	8*	12.5
Pelvic radiation therapy	3	4.7
Tamoxifen use (ever)	7	11.0
Median CA125 preoperative (range)	28 (5–1625)	

Diagnosed based on tissue from		
Aspiration biopsy	31	48.4
Hysteroscopic biopsy	8	12.5
Dilatation and curettage	2	3.1
Ultrasound guided biopsy	1	1.6
Postoperative histology	18	28.1
Other	4	6.3

Clinical FIGO Stage (2009)		
Stage I	42	65.6
Stage II	1	1.6
Stage III	7	10.9
Stage IV	14	21.9

* Some patients had multiple malignancies in their medical history.

† Other malignancies: Brain tumor (n = 1), lung tumor (n = 2), oropharynxcarcinoma (n = 1), skin tumor (not melanoma) (n = 4).

Treatment, histological and survival characteristics are described in Table 2. Initial treatment was for 53 (82.8 %) patients with curative intent and nine patients (17.2 %) received palliative treatment, and two (3.1 %) best supportive care. Treatment details are outlined in Table 2. Of the 53 patients treated with curative intent, 37 underwent staging surgery according to protocol (see supplementary table 1), 11 had cytoreductive surgery. For the remaining five patients, a decision was made to not forego staging procedures, due to factors such as advanced age and/or comorbidities and thus only perform a hysterectomy and bilateral salpingo-oophorectomy. Based on pathological staging, 11 cases (17 %) were upstaged, mostly because of positive lymph nodes to stage III (not shown). After surgery, 11 patients (19 % of the total 57 patients who had surgery) had perioperative complications, and 12 patients (21 % of the total 57 patients who had surgery) postoperative complications within 30 days after surgery. One patient experienced a fatal complication following surgery, with a pulmonary embolism likely being the cause of death (supplementary table 2).

**Table 2:** Treatment, histological, and survival characteristics.

	**Characteristics**	**N = 64**	**%**
Treatment	**Treatment intent**		
	Curative	53	82.8
	Palliative care	9	14.1
	Best supportive care	2	3.1
	**Surgery in patients with curative intent**	**53**	**82.8**
	Staging*	37**	54.7
	Median no. of pelvic lymph nodes removed (range)	23 (6–54)	
	Median no. of *para*-aortic lymph nodes removed (range)	5 (0–21)	
	Cytoreductive surgery	11	17.2
	Complete cytoreductive surgery	10†	15.6
	Optimal cytoreductive surgery	1	1.6
	Hysterectomy and bilateral salphingo-oophorectomy only	5	7.8
	**(Neo)adjuvant chemotherapy in patients with curative intent**	**22**	**34.4**
	Neo-adjuvant + adjuvant	7	10.9
	Adjuvant only	15	23.4
	**Adjuvant radiotherapy in patients with curative intent**	**27**	**46.6**
	Vaginal brachytherapy only	13	20.3
	External beam pelvic radiation only	8	12.5
	External beam vaginal brachytherapy and pelvic radiation	5	7.8
	External beam pelvic and *para*-aortic radiation	1	1.6
	**Surgery in patients receiving palliative care**	**4**	**4.7**
	Incomplete cytoreductive surgery	1	1.6
	Hysterectomy and bilateral salphingo-oophorectomy only	3	3.1
	**Palliative chemotherapy**	**6**	**9.4**
	**Palliative radiotherapy**	**2**	**3.1**

HIstological	Median tumor size in mm (range)	70 (8–400)	
	Pathological FIGO Stage (2009)		
	Stage I	32	50.0
	Stage II	1	1.6
	Stage III	13	20.3
	Stage IV	18	28.1
	Lymphovascular space invasion		
	No	37	57.8
	Yes	20	31.3
	Unknown	7	10.9
	Epithelial component		
	Serous	37	57.8
	Endometrioid	9	14.1
	Clear cell	1	1.6
	Undifferentiated	9	14.1
	Unknown	8	12.8
	Mesenchymal component		
	Homologous	31	48.4
	Heterologous	24	37.5
	Unknown	9	14.1
	Sarcoma dominance (>50 % sarcoma)		
	No	28	43.8
	Yes	25	39.1
	Unknown	11	17.2

Survival	Progression of disease		
	Yes	40	62.5
	No	17	26.6
	No, but died due other reason	7	10.9
	Vital status		
	Alive	20	31.3
	Death due to progression of disease	37	57.8
	Death due to other reason than progression of disease	7	10.9

* Staging was performed using an open procedure in 27 patients (73% of 37), while the remaining 10 patients (27%) underwent a minimally invasive procedure.

** two patients had a second surgery for completion of the staging.

† two patients were initially planned for staging.

Seven patients with clinical stage IV disease received neo-adjuvant chemotherapy, followed by interval cytoreductive surgery, and adjuvant chemotherapy. Fifteen patients received primary surgery followed by adjuvant chemotherapy. Chemotherapy was administered as 4–6 cycles of three-weekly carboplatin/paclitaxel. Three patients opted out of adjuvant chemotherapy.

Of the 53 patients treated with curative intent 27 (50.1 %) received adjuvant radiotherapy. Almost half of them received vaginal brachytherapy only with curative treatment intent (n = 13). Eight patients had external beam radiation therapy only and five a combination of vaginal brachytherapy and external beam radiation therapy. One patient received pelvic and *para*-aortic radiation. Six patients declined radiotherapy.

Nine patients received palliative care and two best supportive care. Initially, two patients started treatment with curative intent, but the extent of their disease led to a palliative resection. One patient received palliative chemotherapy and radiotherapy after surgery. Two patients had a palliative hysterectomy and bilateral salpingo-oophorectomy to alleviate vaginal bleeding, followed by palliative chemotherapy in one of them. Four patients received palliative chemotherapy only, and one patient received palliative radiotherapy only. Two patients were treated with the best supportive care, meaning that these patients did not receive any tumor-specific procedures, but treatment for focusing solely on comfort.

Tumor diameter ranged from 8-400 mm, with 45.3 % having ≤ 50 % myometrial involvement. Lymphovascular space invasion (LVSI) was present in 31.3 % of cases. The epithelial/carcinoma component was predominantly serous (58 %), and the mesenchymal/sarcoma pattern was homologous in 48 %. Sarcoma dominance (>50 % sarcoma component) was noted in 39 % of patients (table 2).

After initial treatment, forty patients (63 %) had progression of disease with a median of 8 months (range 0–39 months) after initial diagnosis. In all cases except for two, progression occurred within two years. Second-line treatment was personalized, considering the patient's individual characteristics and preferences and the extent of the recurrence. The survival analysis showed a median progression-free survival of 13 months (range 1–99 months). Median overall survival was 20 months (range 1–99).

**Fig. 1:**
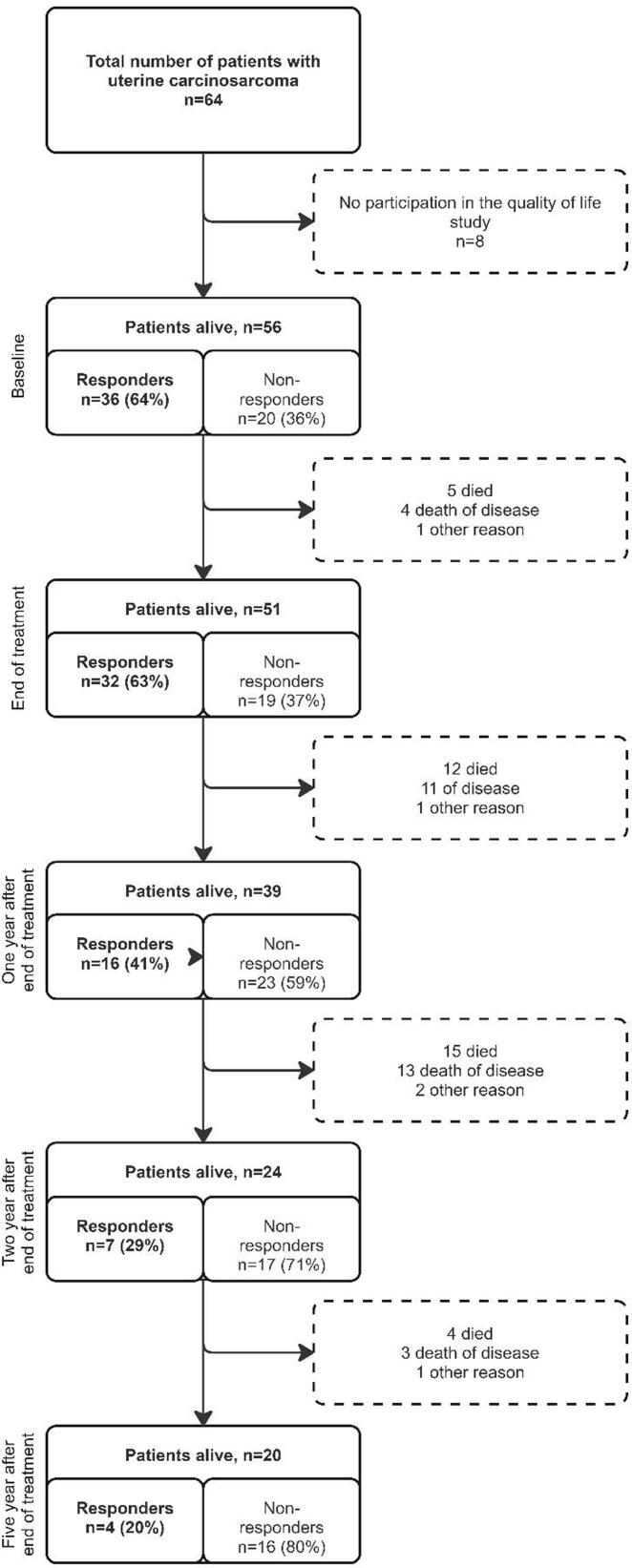
Flowchart of inclusions for QoL-questionnaries with responders, non-responders and survival data.

**Fig. 2:**
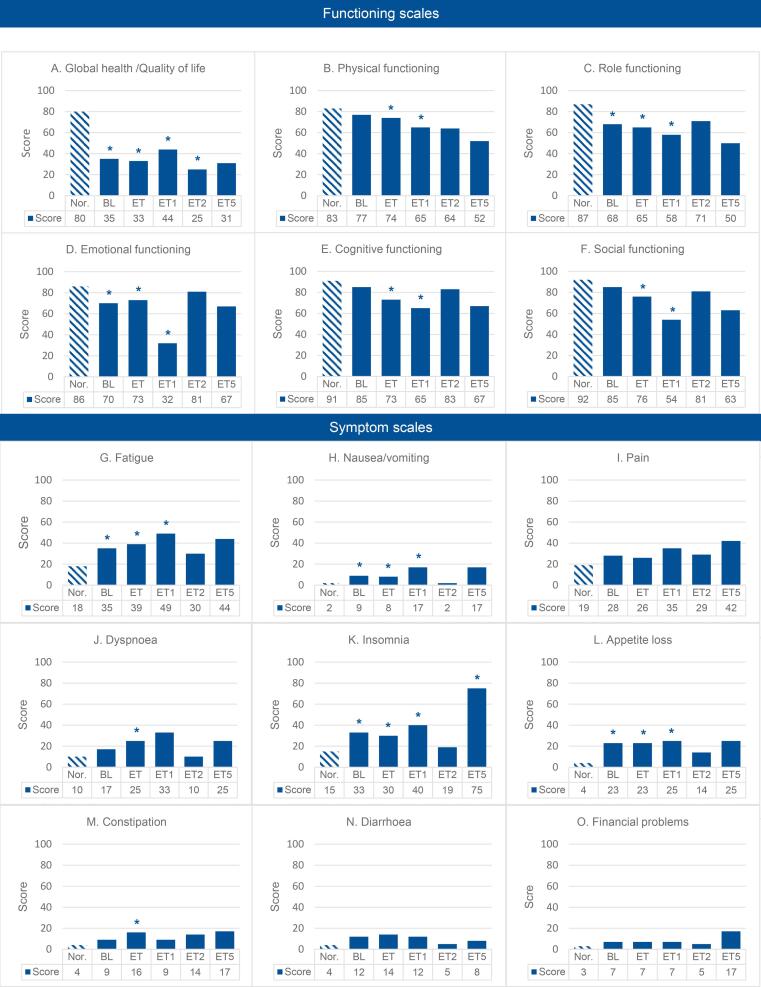
Overview of EORTC QLQ-C30 scores compared with age- and gender-matched normative data from De Ligt et al., 2023. The first bar represents the mean of the normative data, the remaining bars depict the mean scores at each time point for the study population. Bars marked with an asterisk indicate a statistically significant difference between the study population's mean and the normative data mean. In functioning domains, higher scores reflected better quality of life. In symptom domains, higher scores signified more severe symptoms. Definition of abbreviations: Nor. = normative data; BL = baseline; ET = End of treatment; ET1 = One year after treatment; ET2 = Two years after treatment; ET5 = Five years after treatment.

**Fig. 3:**
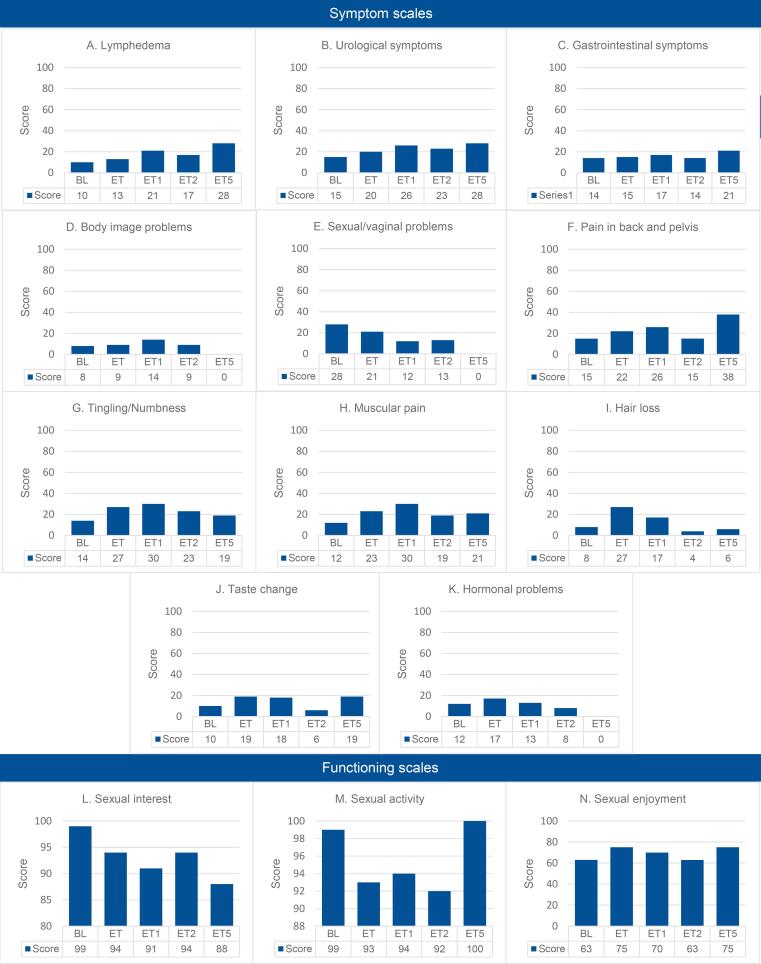
Overview of EORTC EN24 scores. The bars depict the mean scores at each time point for the study population. In functioning domains, higher scores reflected better quality of life. In symptom domains, higher scores signified more severe symptoms. Definition of abbreviations: Nor. = normative data; BL = baseline; ET = End of treatment; ET1 = One year after treatment; ET2 = Two years after treatment; ET5 = Five years after treatment.

Among the 56 patients in QoL assessments, baseline response rate was 64 % (n = 36) (Fig. 1). Response rates declined over time. Data from QLQ-C30 in our study were compared to data from a matched group of the normal Dutch population women provided by De Ligt et al. (de Ligt et al., 2023) revealing that QoL remains compromised even one year after treatment has ended (Fig. 2;supplementary table 3). While symptom domains are only mildly impacted, except for fatigue, all functioning domains (global health, physical, role, emotional, cognitive, and social) show significant deterioration one year after treatment. The EN24 questionnaire reveals a similarly low QoL across several domains immediately following the end of treatment, persisting in the years afterwards (Fig. 3, supplementary table 4). Most affected domains at the end of treatment include urological, sexual/vaginal issues, back and pelvic pain, tingling/numbness, muscle pain, and hair loss, likely due to treatment effects. Except for hair loss, theses scores remained low one year after treatment, which could partly be attributed to the recurrence of disease, as many patients experience recurrence within the two year treatment. Moreover, lymphedema became more pronounced one year after treatment than immediately following treatment.

Outcomes of the survival analysis are listed in Table 3. On univariable analysis surgical FIGO stage, surgery, chemotherapy and radiotherapy were statistically significant. All these variables, except for radiotherapy, remained significant prognostic factors upon multivariable analysis.

**Table 3:** Uni- and multivariable analysis for overall survival.

Table 3 **Uni- and multivariable analysis for overall survival**					
	**Median OS in months**	**Univariable analysis**		**Multivariable analysis**	
		**Hazard ratio** **(95 % CI)**	**p-value**	**Hazard ratio** **(95 % CI)**	**p-value**
**Age**					
<69 years	18	1			
≥ 70 years	34	0.59 (0.31–1-16)	0.118		

**WHO**					
0	34	1			
1	18	1.41 (0.65–3.06)			
2-3	24	1.15 (0.40–3.32)	0.668		

**Moment of diagnosis of UCS**					
Preoperative	15	1			
Postoperative	34	0.57 (0.26–1.26)	0.152		

**Postoperative FIGO Stage**					
I-II	51	1		1	
III	21	1.69 (0.71–4.0)		7.09 (1.91–26.35)	
IV	10	3.24 (1.55–6.78)	**0.004**	8.36 (2.82–24.76)	<**0.001**

**Tumor size**					
<7.0 mm	31	1			
≥7.0 mm	34	0.9 (0.45–1.90)	0.830		

**Myometrial invasion**					
No	NR	1			
<0.5	21	1 (0.89–4.3)			
≥ 0.5	51	1.0 (0.3–3.4)	0.21		

**LVSI**					
No	34	1			
Yes	19	1.1 (0.2–2.3)	0.82		

**Epithelial component**					
Serous	31	1			
Endometroid	24	1.5 (0.6–3.5)			
Other	21	0.9 (0.3–2.5)	0.641		

**Mesenchymal component**					
Homologous	24	1			
Heterologous	34	0.88 (0.39–1.69)	0.57		

**Sarcoma dominance**					
No	34	1			
Yes	15	1.7 (0.8–3.6)	0.123		

**Surgery**					
No	3	1		1	
Yes	31	0.1 (0.04–0.24)	**<0.001**	0.03 (0.01–0.143)	**<0.001**

**Chemotherapy**					
No	31	1		1	
Yes, neo- and adjuvant	10	2.49 (0.84–7.39)		0.47 (0.10–2.14)	
Yes, adjuvant	N.R.	0.68 (0.28–1.69)		0.17 (0.05–0.60)	
Yes, palliative	9	2.61 (0.97–7.03)	**0.039**	0.36 (0.08–1.53)	**0.035**

**Radiotherapy**					
No	17	1		1	
Yes, curative setting	21	0.83 (0.42–1.64)		1.43 (0.57–3.62)	
Yes, palliative setting	2	7.4 (1.59–34.8)	**0**.**004**	3.73 (0.50–27.67)	0.355

Definition of abbreviation: OS = overall survival, LVSI = lymph-vascular space invasion NR = not reached, UCS uterine carcinosarcoma.

## 4 Discussion

### 4.1 Summary of main results

We performed a prospective analysis of data from 64 patients with UCS in a tertiary referral center. We described the effect of disease and treatment on QoL of patients with UCS using the EORTC QLQ-C30 and EN24 questionnaires. Furthermore, we disclosed prognostic factors of UCS on the survival.

QoL of patients is influenced by both disease symptoms and treatment-related factors. Since UCS shares similar symptoms and treatment procedures with high-risk endometrial cancer, the most impacted domains in QoL in our study are physical functioning, cognitive function (memory problems), emotional functioning (anxiety/depression), fatigue, insomnia, lymphedema, neurological and gastrointestinal symptoms, tingling/numbness, and pain (Gil-Ibanez et al., 2023). The QoL of patients can be affected by symptoms of the disease and subsequent treatment procedures. For UCS, symptoms and treatment are comparable to that of high-risk endometrial cancer, explaining the similarities in effect on QoL in patients with UCS compared to other types of endometrial cancer.

To minimize the effect of the treatment on QoL opting for less invasive procedures, such as laparoscopic or robotic staging instead of laparotomy, and sentinel lymph node procedures rather than full lymphadenectomy, could be beneficial (Ferguson et al., 2018, Bjerre Trent et al., 2023, Angioli et al., 2013). In our cohort the majority of the patients underwent laparotomic staging, as this aligned with guideline recommendations during the initial years of our study. Due to the relatively small sample size, we were unable to stratify our QoL analysis based on surgical approach, However, it is likely that patients who underwent laparotomy experienced a greater impact on QoL compared to those who had a minimally invasive procedure. Currently, minimally invasive surgery is the preferred approach for high-risk endometrial cancer, including UCS, when feasible which is likely to have positive effects on the QoL. Regarding lymph node procedure, current guidelines for uterine carcinosarcoma still favor lymphadenectomy over sentinel node procedures (Concin et al., 2021, Abu-Rustum et al., 2024, Restaino et al., 2023). Nevertheless, emerging studies suggest that sentinel node procedures could be a safe and less invasive alternative, even for high-risk endometrial cancers like UCS (Zammarrelli et al., 2022, Holtzman et al., 2023). The main advantage of sentinel node procedures over lymphadenectomy is the reduced risk of lymphedema, which can significantly improve QoL (Bjerre Trent et al., 2023). While these considerations apply to other high-risk endometrial cancers, certain factors make prioritizing QoL especially critical for UCS patients..

The relatively poor QoL observed one year after treatment in our study is in contrast with previous research on endometrial cancer, which generally suggest that QoL returns to normal within four to six months (Shisler et al., 2018, Ferguson et al., 2018, Herling et al., 2016, Berretta et al., 2015, Arms et al., 2015, Ferrandina et al., 2014, Honerlaw et al., 2016, de Boer et al., 2015, Salehi et al., 2018). These studies included both low- and high-risk endometrial carcinoma with varying degrees of treatment intensity. While direct comparisons between these studies and patients with UCS are limited, the findings suggest that the aggressiveness of UCS and the extensive treatments given have a more pronounced impact on QoL. Remarkably, in this cohort 37.5 % of the patients with UCS had a history of another malignancy, with breast cancer being the most prevalent (23.4 % of the total study population) and non-invasive skin cancer second (6.4 %). The number patients with a medical history of cancer is higher compared to other studies, and also compared other histological types of endometrial cancer which more varies between 11–26 % (Cantrell et al., 2012, Felix et al., 2011, Maiorano et al., 2024). Tamoxifen and pelvic radiation are established risk factors for developing endometrial cancer, therefore medical history of breast cancer and subsequent use of tamoxifen, and bladder and/or rectal cancer with pelvic radiation is commonly observed in patients with endometrial cancer.

Although not accounted for in the QoL analysis, a prior medical history may have already impacted these patients' QoL which is reflected in the relatively poor QoL-scores observed. The prolonged QoL impairment years after treatment may be attributed to disease recurrence, as our data indicate that nearly all recurrences occur within two years of treatment.

The impact on QoL is particularly meaningful given the poor prognosis of patients with UCS. In our cohort, median progression-free survival was 13 months, and overall survival was 20 months. In our study, we chose a unified starting point for the survival timeline to ensure consistency, even for patients who did not receive any treatment. Nonetheless, a progression-free survival of 13 months and an overall survival of 20 months highlight the poor prognosis of UCS patients.. Prognostic factors provide insights in the gain of life expectancy. In our survival analysis stage and treatment were significant prognostic factors, consistent with previous literature. Yet, patients in our dataset with early stage and treatment still experienced recurrence of disease. Therefore, it is crucial to consider side effects and impact on QoL in relation to life expectancy. Previous literature has shown that more than half of patients prioritize QoL over survival gains among a variety of types of advanced-stage cancer (Kypriotakis et al., 2016, Brundage et al., 2023, Fleischer et al., 2023, Yong et al., 2023). Yong et al. (Yong et al., 2023) have found that patients favored improvements in QoL, particularly in physical functioning and pain management, over an additional year of survival. These preferences can vary among patients based on factors such as cultural background, values, and age, and should be carefully considered by treating clinicians.

### 4.2 Results in Context of Published literature

While numerous studies have explored the QoL in endometrial cancer survivorship, none have specifically focused on patients with UCS. As treatment of UCS is like that of other high-risk endometrial cancers, a comparable impact on QoL was expected. In our study, QoL measures could not be directly linked to variables such as the type of surgery, chemotherapy, or age due to the limited sample size. However, the affected domains correlate with the treatments patients received and their known effects on QoL. For example, our cohort exhibited an increase in lymphedema, a known complication following pelvic lymphadenectomy and radiotherapy, one year after treatment, consistent with its occurrence between six months and several years post-treatment (Bjerre Trent et al., 2023). Differences in QoL after laparoscopy versus laparotomy were observed, particularly in physical and emotional functioning and body image, though these differences typically diminished six months post-surgery (Ferguson et al., 2018, Salehi et al., 2018, Janda et al., 2010, Zullo et al., 2005). Approximately one third of our patients received chemotherapy. A decline in QoL-scores on domains of chemotherapy-related symptoms such as tingling/numbness, hair loss, and cognitive impairment (Westin et al., 2016, Deprez et al., 2012, Was et al., 2022) were also observed in our study. Additionally, adjuvant chemotherapy and/or radiotherapy, particularly when combined, were associated with a lower QoL (de Boer et al., 2015, Westin et al., 2016). Overall, our findings suggest that the extensive treatment regimens for UCS in combination with the poor prognosis significantly impacted patients' QoL.

### 4.3 Strengths and weakness

Our study is the first to explore the QoL in patients with UCS, a crucial area of research given the poor prognosis of UCS. Additionally, by conducting a longitudinal analysis with follow up up to five years, we provided valuable data on the long-term QoL of survivors. Administering questionnaires at specific time points helped minimize recall bias, allowing us to accurately assess QoL at the intervals. However, the primary limitation of our study is the potential bias due to loss to follow up. This is partly due to patient mortality over time, which introduces survival bias. Furthermore, poorer response rates among the survivors of 29 % at two years and 20 % at five years after treatment with a risk for confounding. It is thinkable that patients with many symptoms, and thus a poor QoL were less likely to respond because they are limited due to this poor QoL. Consequently, the reported outcomes may reflect the QoL of those who survived and were doing relatively well, suggesting that the true QoL could be lower. Lastly, our cohort was relatively small, preventing us from making comparisons between groups based on clinical or treatment characteristics.

### 4.4 Implication for practice and future research

Although UCS is a rare histological subtype of endometrial cancer, it’s proportion among endometrial cancer cases is increasing (Matsuo et al., 2018). This underscores the need for further research. The clinical significance of QoL in patients with UCS is particularly important due to their poor prognosis. This raises the question whether the benefits of extensive treatment outweigh the associated harms. This is especially important when some patients may prefer to avoid aggressive interventions even at the cost of a shorter life expectancy. Opting for less invasive procedures, such as laparoscopic or robotic staging instead of laparotomy, and sentinel lymph node procedures rather than full lymphadenectomy, could improve post-treatment QoL. Minimally invasive surgery is already the preferred approach when feasible, including for high-risk endometrial cancer. However, in uterine carcinosarcoma, lymphadenectomy remains favored over sentinel node procedures in the current guidelines (Concin et al., 2021, Abu-Rustum et al., 2024, Restaino et al., 2023), though there are studies indicating that sentinel node procedures could be a safe and less invasive alternative for lymphadenectomy even in high-risk endometrial cancers such as UCS. Such decisions should be made through shared decision-making.

Future studies should focus on patients' preferences and incorporating QoL considerations into treatment plans. Studies exploring alternative procedures to mitigate complications should also examine their impact on QoL. It is plausible that a lower complication rate would correlate with improved QoL. Additionally, identifying prognostic and predictive markers to guide clinicians in determining which patients might truly benefit from intensive treatment.

## 5 Conclusion

This study highlights the significant impact of disease and extensive treatment on QoL in patients with UCS. QoL remains compromised long after treatment, with notable declines in physical, emotional, and social functioning, as well as persistent symptoms like fatigue, pain, and lymphedema. The findings underscore the need for a balanced approach in treatment decisions, particularly given the poor prognosis associated with this cancer type. The study also emphasizes the importance of integrating QoL considerations into clinical practice and further research to better tailor treatment strategies.

## CRediT authorship contribution statement

**Eveline N.B. Pham:** Writing – original draft, Software, Resources, Methodology, Investigation, Formal analysis, Data curation, Conceptualization. **Caroline B. van den Berg:** Writing – review & editing, Supervision, Methodology, Conceptualization. **Ingrid Boere:** Writing – review & editing. **Vera de Geus:** Writing – review & editing, Resources. **Juanita A. Haagsma:** Writing – review & editing, Visualization. **Marianne Maliepaard:** Writing – review & editing, Resources, Project administration. **Jan Willem M. Mens:** Writing – review & editing, Conceptualization. **Floris H. Groenendijk:** Writing – review & editing, Conceptualization. **Heleen J. van Beekhuizen:** Writing – review & editing, Supervision, Methodology.

## Declaration of competing interest

The authors declare that they have no known competing financial interests or personal relationships that could have appeared to influence the work reported in this paper.
