# Supplementary material for: Quality of life and survival in patients with uterine carcinosarcoma: A tertiary center observational study
Source: Gynecol Oncol Rep. 2025 Jan 17;57:101679. doi: 10.1016/j.gore.2025.101679 (PMC11788790; doi:10.1016/j.gore.2025.101679)
Supplement: Supplementary Data 1 [file mmc1.pdf]

Supplementary table 1 Treatment protocol 2017-2022 of uterine carcinosarcoma at the Erasmus MC, Rotterdam, the Netherlands.

Stage	Surgery	Chemotherapy	Radiotherapy	If staging/radical hysterectomy is not feasible
IA	Complete staging with lymph node dissection	None	None	External radiotherapy if LVSI+
IB	Complete staging with lymph node dissection	None	Brachytherapy	External radiotherapy if LVSI+ and/or deep myometrial invasion
II	Radical hysterectomy and complete staging with lymph node dissection	None	Brachytherapy	External RT if LVSI+, deep myometrial invasion, cervical involvement and/or non-radical removal; possibly (brachytherapy or external) boost
IIIA	Cytoreductive surgery* with lymph node dissection	6 cycles of paclitaxel/ carboplatin	External radiotherapy	Chemo- and radiotherapy
IIIB	Cytoreductive surgery with lymph node dissection	6 cycles of paclitaxel/ carboplatin	External RT + consider external or brachytherapy boost	Chemo- and radiotherapy
IIIC	Cytoreductive surgery* and consider lymph node dissection	6 cycles of paclitaxel/ carboplatin	External RT + consider boost in case of non-radical removal	Chemo- and radiotherapy
IV curative intent	(Interval) cytoreductive surgery	Induction chemotherapy with paclitaxel/ carboplatin followed by surgery and 3 additional cycles, or 6 cycles after primary surgery	External RT unless disease spreads beyond the pelvis, groin, or para-aortic nodes	Postoperative diagnosis: 6 cycles of paclitaxel/carboplatin and external radiotherapy
IV best supportive care	None or to procedure needed to alleviate symptoms	Palliative chemotherapy	Palliative radiotherapy	Not applicable

*=Radical hysterectomy in cases with cervical involvement.

Definition of abbreviation: LVSI=lymph-vascular space invasion
