# Supplementary material for: Quality of life and survival in patients with uterine carcinosarcoma: A tertiary center observational study
Source: Gynecol Oncol Rep. 2025 Jan 17;57:101679. doi: 10.1016/j.gore.2025.101679 (PMC11788790; doi:10.1016/j.gore.2025.101679)
Supplement: Supplementary Data 2 [file mmc2.pdf]

Supplementary table 2 Peri- and postoperative complications

Complications	N=64	%
Perioperative complications	11*	17.2
Blood loss > 1L	3	4.7
Bladder lesion	1	1.6
Urethral lesion	1	1.6
Bowel injury	2	3.1
Wound infection	1	1.6
Admission to intensive care	1	1.6
Vascular lesion	2	3.1
Spleen lesion	1	3.1
Subcutaneous emphysema	1	3.1
Postoperative complications	12*	18.8
Pneumonia	1	1.6
Sepsis	2	3.1
Intra-abdominal abscess	1	1.6
Respiratory insufficiency	2	3.1
Lung embolism	1*	1.6
Delirium	2	3.1
Relaparotomy	3	5.9
Gastroparesis/ileus	2	3.1
Fever of unknown origin	1	1.6
Abdominal wound dehiscence	2	3.1
Kidney insufficiency	1	1.6
Urinary incontinency	1	1.6

* some patients have multiple complications

† Patient died due to lung embolism
