# Supplementary material for: Quality of life and survival in patients with uterine carcinosarcoma: A tertiary center observational study
Source: Gynecol Oncol Rep. 2025 Jan 17;57:101679. doi: 10.1016/j.gore.2025.101679 (PMC11788790; doi:10.1016/j.gore.2025.101679)
Supplement: Supplementary Data 3 [file mmc3.pdf]

Supplementary table 3. Mean scores of EORTC QLC-C30 questionnaires

		Mean scores (SD)										
		**Normative data**	**Baseline**	**p-value ***	**End of treatment**	**p-value ***	**One year**	**p-value ***	**Two years**	**p-value ***	**Five years**	**p-value ***
	Number of responders		36		32		16		7		4	
	Number of patients alive		56		51		39		24		20	
Functioning domain	Global health	80.2 (3.17)	34.5 (22.6)	**<0.001**	32.6 (22.5)	**<0.001**	43.8 (27.0)	**<0.001**	25.0 (18.0)	**<0.001**	31.3 (32.2)	0.056
	Physical	83.8 (18.7)	77.1 (19.6)	0.056	74.4 (22.6)	**0.021**	64.6 (25.1)	**0.026**	63.8 (23.0)	0.109	51.7 (39.0)	0.240
	Role	86.7 (2.3)	68.0 (31.0)	**0.001**	65.1 (31.5)	**<0.001**	58.3 (37.0)	**0.008**	71.4 (23.0)	0.129	50.0 (43.0)	0.186
	Emotional	86.2 (2.7)	69.2 (21.6)	**<0.001**	72.9 (28.4)	**0.009**	61.5 (25.1)	**0.001**	81.0 (23.9)	0.576	66.7 (37.9)	0.376
	Cognitive	91.2 (0.3)	84.7 (23.7)	0.121	72.9 (30.2)	**0.001**	64.6 (31.5)	**0.004**	83.3 (25.5)	0.446	66.7 (47.1)	0.375
	Social	92.2 (0.9)	84.7 (23.7)	0.075	76.0 (32.5)	**0.007**	54.2 (34.2)	**<0.001**	81.0 (37.8)	0.464	62.5 (47.9)	0.303
Symptoms domain	Fatigue	17.9 (2.4)	34.5 (28.7)	**0.002**	39.2 (33.7)	**<0.001**	49.3 (27.5)	**<0.001**	30.2 (23.8)	0.220	44.4 (52.1)	0.383
	Nausea/ vomiting	1.5 (0.3)	9.0 (20.6)	**0.042**	8.3 (20.2)	0.059	16.7 (27.9)	**0.046**	2.4 (6.3)	0.726	16.7 (33.3)	0.429
	Pain	18.6 (1.5)	27.5 (30.7)	0.101	25.5 (32.8)	0.230	35.4 (34.4)	0.070	28.6 (34.3)	0.470	41.7 (50.0)	0.424
	Dyspnea	9.9 (1.7)	17.1 (29.0)	0.157	25.0 (30.5)	**0.007**	33.3 (36.5)	0.128	9.5 (16.3)	0.190	25.0 (50.0)	0.815
	Insomnia	15.1 (1.7)	33.3 (32.4)	**0.003**	30.2 (32.1)	**0.010**	39.6 (34.9)	**0.013**	19.0 (17.8)	0.586	75.0 (31.9)	**0.033**
	Appetite loss	4.2 (0.9)	22.5 (30.5)	**0.001**	22.9 (32.2)	**0.002**	25.0 (33.3)	**0.025**	14.3 (26.2)	0.346	25.0 (50.0)	0.466
	Constipation	4.1 (0.9)	9.0 (18.7)	0.138	15.6 (29.3)	**0.029**	9.0 (18.7)	0.313	14.3 (17.8)	0.891	16.7 (19.2)	0.281
	Diarrhea	4.3 (0.6)	11.7 (22.5)	0.063	13.5 (29.2)	0.074	11.7 (22.5)	0.207	4.8 (12.6)	0.916	8.3 (16.7)	0.663
	Financial problems	3.3 (0.7)	7.4 (18.0)	0.196	7.3 (22.0)	0.300	7.4 (8.0)	0.060	4.8 (12.6)	0.227	16.7 (33.3)	0.481

In functioning domains, higher scores reflected better quality of life. In symptom domains, higher scores signified more severe symptoms.
* p-value compared with normative data, matched with age-group and gender from the study of De Ligt et al. (21)
