# Supplementary material for: Quality of life and survival in patients with uterine carcinosarcoma: A tertiary center observational study
Source: Gynecol Oncol Rep. 2025 Jan 17;57:101679. doi: 10.1016/j.gore.2025.101679 (PMC11788790; doi:10.1016/j.gore.2025.101679)
Supplement: Supplementary Data 4 [file mmc4.pdf]

Supplementary table 4. Mean scores of EORTC EN24 questionnaires

		Mean scores (SD)				
		Baseline	End of treatment	One year after end of treatment	Two year after end of treatment	Five years after end of treatment
	Number of responders	36	31	21	7	4
	Number of patients alive	56	51	39	24	20
Symptom domains	Lymphedema	10.1 (17.4)	12.9 (15.4)	20.8 (18.7)	16.7 (23.4)	28.1 (35.9)
	Urological	14.6 (15.1)	20.0 (15.2)	25.9 (19.6)	22.9 (20.7)	28.1 (23.1)
	Gastrointestinal	14.2 (11.9)	15.2 (15.2)	16.7 (14.8)	14.2 (15.1)	21.3 (25.0)
	Body image problems	8.3 (17.9)	8.5 (13.8)	14.3 (20.3)	9.4 (16.1)	0 (0)
	Sexual/vaginal problems	27.5 (41.2)	20.8 (24.0)	11.7 (13.9)*	12.5 (4.8)‡	0 (n.a.)§
	Pain in back and pelvis	14.6 (21.0)	21.8 (24.8)	26.2 (27.9)	14.6 (24.9)	37.5 (32.3)
	Tingling/numbness	13.9 (25.0)	26.6 (28.1)	29.8 (28.1)	22.9 (22.5)	18.8 (37.5)
	Muscular pain	11.8 (16.4)	22.6 (26.1)	29.8 (28.1)	18.8 (18.8)	31.3 (37.5)
	Hair loss	8.3 (20.7)	27.4 (33.1)	16.7 (29.9)	4.2 (9.7)	6.3 (12.5)
	Taste change	10.4 (0.2)	19.4 (25.6)	17.9 (28.7)	6.3 (15.5)	18.8 (37.5)
	Hormonal problems	11.5 (16.5)	16.9 (21.3)	13.1 (20.0)	8.3 (12.3)	0 (0) §
Functioning domains	Sexual interest	98.6 (5.8)	93.5 (14.4)	90.5 (14.7)	93.8 (11.3)	87.5 (25.0)
	Sexual activity	98.6 (5.8)	92.5 (10.0)	94.0 (10.9)	91.7 (12.3)	100 (12.5)
	Sexual enjoyment	62.5 (17.7)	75.0 (15.8)†	70.0 (20.9)*	62.5 (14.4)‡	75.0 (n.a.)§

In functioning domains, higher scores reflected better quality of life. In symptom domains, higher scores signified more severe symptoms.

* mean based on 5 responders

† mean based on 6 responders

‡ mean based on 4 responders

§ Number based on one responder

Definition of abbreviations: SD=standard deviation, n.a.=not applicable
